# Dulcitol/Starch Systems as Shape-Stabilized Phase Change Materials for Long-Term Thermal Energy Storage

**DOI:** 10.3390/polym16223229

**Published:** 2024-11-20

**Authors:** Martyna Szatkowska, Kinga Pielichowska

**Affiliations:** Department of Biomaterials and Composites, Faculty of Materials Science and Ceramics, AGH University of Krakow, Al. Mickiewicza 30, 30-059 Kraków, Poland; szatkowska@agh.edu.pl

**Keywords:** shape stable phase change materials (SSPCM), dulcitol, sugar alcohol, long-term thermal energy storage

## Abstract

In recent years, there has been an increasing interest in phase change materials (PCM) based on dulcitol and other sugar alcohols. These materials have almost twice as large latent heat of fusion as other organic materials. Sugar alcohols are relatively cheap, and they can undergo cold crystallization, which is crucial for long-term thermal energy storage. The disadvantage of dulcitol and other sugar alcohols is the solid–liquid phase transition. As a result, the state of matter of the material and its volume change, and in the case of materials modified with microparticles or nanoparticles, sedimentation of additives in liquid PCM can occur. In this study, we obtained shape-stable phase change materials (SSPCM) by co-gelation of starch and dulcitol. To characterize the samples obtained, differential scanning calorimetry (DSC), step-mode DSC, thermogravimetric analysis (TGA), Fourier transform infrared spectroscopy (FTIR), and scanning electron microscopy (SEM) were used, and they were also used to test for shape stabilization. The results show that the obtained systems have great potential as shape-stabilized phase change materials. The sample dulcitol/starch with a 50:50 ratio exhibited the highest heat of cold crystallization, up to 52.90 J/g, while the heat of melting was 126.16 J/g under typical DSC measuring conditions. However, depending on the applied heating program, the heat of cold crystallization can even reach 125 J/g. The thermal stability of all compositions was higher than the phase change temperature, with only 1% mass loss occurring at temperatures above 200 °C, while the phase change occurred at a maximum of 190 °C.

## 1. Introduction

Sugar alcohols have attracted more and more attention in recent years as organic phase change materials (PCM) for long-term thermal energy storage. The particular advantage of these materials is the low price but the high value of latent heat of phase transition [1,2,3,4,5,6,7,8].

The ability to store energy in PCMs has a major impact on the application areas. Some applications may require energy storage for up to several months. The inadequate long-term stability of the storage materials is attributed to two factors: the poor stability of the material properties and/or the corrosion between the PCM and the container [9,10].

Recently, the main goal of intensive research has been the development of materials capable of long-term thermal energy storage. This effect can be achieved in materials able to thermal energy storage during heating, but during cooling they do not undergo crystallization and release thermal energy, as they are a supercooled liquid. Unfortunately, such materials are metastable, and there is always a risk of spontaneous crystallization, which could prematurely release the latent heat during cooling [11,12,13]. Turunen et al. [11] investigated the erythritol/mannitol mixture in an ionically cross-linked poly(vinyl alcohol) (PVA) scaffold on thermal properties, in particular cold crystallization. A composition consisting of 80 wt% PCM, 10 wt% PVA, and 10 wt% sodium citrate (SC) demonstrated both high melting enthalpy (176 J/g) and shape stability, making it suitable for larger scale and leakage-free applications [11]. A novel approach employing the process of cold crystallization was introduced by Li et al. [14], who, in their work, modified the supercooling stability of sugar alcohols (PCMs) by introducing alkali hydroxides. The material remained in a liquid state only after initiating cold crystallization by adding crystallization seeds or mechanical deformation that resulted in heat release [14].

One way to test the long-term performance of PCM material is by placing PCM in containers inside a temperature-controlled oven when exposed to a constant temperature above their melting point. This test allows us to check the impact on the thermal characteristics of PCMs with time. Behzadi et al. [15] demonstrated that paraffin-based PCM experiences an irreversible physical transformation that modifies its thermal properties; in contrast, the more stable ester mixture maintained its mass and remained thermally stable throughout the heating period.

Shao et al. [5] investigated the thermal stability of dulcitol after multicycle in an atmosphere of air and nitrogen. In a protective atmosphere, a decrease in thermal stability of 47.2% was recorded after 250 cycles, while in an air atmosphere, it decreased by 58.5% after 70 cycles. The use of a protective atmosphere does not cause the oxidation of sugar alcohols to aldehydes. In the literature, Shao et al. [16] studied sugar alcohols for 20 h heating in superheat at 5 K to check if the material would undergo the oxidation process. The FTIR result shows a new peak at 2661.1 cm^−1^—stretching vibrations of the C-H bond characteristic for aldehyde groups. Solé et al. [17] and Neuman et al. [17,18]. In contrast, Shao et al. [5] stated that only a small part of the sample was oxidized, and the ketone or aldehyde could not be clearly detected based on the FTIR test.

In our research, we focus particular attention on six sugar alcohols: meso-erythritol (C_4_H_10_O_4_), xylitol (C_5_H_12_O_5_), d-sorbitol (C_6_H_14_O_6_), d-mannitol (C_6_H_14_O_6_), myo-inositol (C_6_H_12_O_6_) and d-dulcitol (C_6_H_14_O_6_). Several of them have the potential to be used in thermal energy storage (TES) applications due to their wide melting point range, high heat of fusion, good thermal stability, and lack of corrosion effect on the metallic environment [19]. Dulcitol is one of the materials that, despite slight overheating, is thermally stable. Both the melting point and the latent heat of fusion of these sugar alcohols tend to degrade with heating and a higher degree of superheat leads to faster degradation [16]. Mesoerythritol and d-dulcitol have high enthalpy (335 J/g and 330 J/g, respectively) [20]. In the literature, the most common studies on meso-erythritol were conducted [20,21,22,23,24]. Dulcitol was tested as a phase change material (substrate) or in mixtures with other sugars [16,21,25,26,27,28,29].

In the literature, various strategies of PCMs shape stabilization can be found in order to reduce the problem of material leakage during phase transformation. Therefore, Wang et al. [30] studied the properties of polyurethane (PU) template-based erythritol/graphite foam composite. The authors used commercially available polyurethane foam, cleaned with sodium hydroxide solution, which additionally allowed the PU wall to have a rough character. The foam was dried and filled with mesophase pitch/multi-walled carbon nanotubes (MWCNTs) slurry. The foam was subsequently heat treated to obtain carbon foams, which were impregnated with meso-erythritol. The samples differed in the content of carbon nanotubes. The higher content of MWCNTs resulted in higher surface roughness and more nucleation sites for heterogeneous nucleation. Additionally, the nanoadditive reduces the size of the pores, which has a positive effect on the flow of material from porous samples (it is lower), while smaller pores result in smaller amounts of infiltrated meso-erythritol (lower enthalpy of phase transformations). The authors indicate that the proposed solution has a positive effect on the disadvantages of meso-erythritol, such as large supercooling, leakage, and low thermal conductivity [30]. Li et al. [31]—meso-erythritol confined in multiwalled carbon nanotubes reinforced silica aerogel. In the first phase, the authors produced a silica aerogel by using a solution of multi-walled carbon nanotubes (MWCNTs) with ethanol, then added tetraethylorthosilicate and deionized water. The solution was brought to an acidic pH with 0.5 M HCl and stirred. The obtained sol solution was treated with ammonia solution to obtain a basic pH and then poured into a vessel to give shape. The additional aerogel was samples made of acid-modified MWCNTs; the initial sample was a silica aerogel without nanoadditives. Meso-erythritol was infiltrated under pressure into the aerogel. Research has shown that the use of acid-modified carbon nanotubes in aerogels generates a porosity that is higher than carbon nanotubes, which generates a higher energy storage capacity. Both aerogels improve the thermal conductivity of the material. The sample with modified nanotubes showed repeatability of the properties after 50 cycles [31]. Pan et al. [32] used graphene aerogel. The authors prepared an aqueous solution of graphene using the modified Hammer method [33]. Then, they added L-ascorbic acid as a reducing agent, mixed the solution, and dried it to obtain a 3D graphene aerogel structure through chemical reduction and self-assembly. The prepared aerogel was freeze-dried and infiltrated with mesoerythritol. In the second case, the authors poured the limited graphene oxide solution into a dish containing copper foam; then, all stages were identical to the previous procedure. The proposed solution achieved a higher amount of stored energy and accurate thermal conductivity, in addition to being equipped with good shape and thermal stability, combined with the meso-erythritol-graphene aerogel configuration [32]. Liu et al. [34] filled alumina porous ceramics with sugar alcohol. The authors produced a cubic alumina porous ceramic made of alumina porous ceramics (APC) slurry consisting of 30 wt% Al(OH)_3_, 50 wt% Al_2_O_3_, 3 wt% ZrO_2_, 2 wt% SiC, 5 wt% Carboxymethylcellulose-Na (CMC-Na) (as thickener), 5 wt% PVA (as binder), and 5 wt% poly(methyl methacrylate) (PMMA). The obtained APC slurry was filled with a commercial melamine resin cube with dimensions of 2 cm × 2 cm × 2 cm and then fired at 1500 °C to remove the organic component, i.e., the resin, and obtain a porous structure. Furthermore, the obtained ceramic foam was modified with 3-aminopropyltriethoxysilane. Two series of samples were obtained, which were infiltrated with three sugar alcohols: erythritol, D-mannitol, and D-dulcitol. Modifying the foam with 3-aminopropyltriethoxysilane improved the compressive strength, which may be beneficial depending on future PCM applications. Filling ceramic foams with alcoholic sugars does not change their structure. The modified structure has better wettability and, therefore, a beneficial effect on subcooling, reducing it by 95% in the case of erythritol. The authors also obtained thermal conductivity, high latent heat, and excellent cycling thermal stability (the enthalpy and temperature of phase changes after 100 cycles were at a level similar to the initial values—difference ~2 J/g) of sugar alcohol composite PCM [34].

In the present paper, dulcitol shapes stabilized with starch by the co-gelation method were investigated as PCMs for long-term thermal energy storage. The thermal properties of SSPCMs were investigated in detail using conventional DSC under various heating and cooling programs and DSC in step mode in order to obtain an optimal program ensuring the highest value of the cold crystallization enthalpy in relation to the melting enthalpy of the material.

## 2. Materials and Methods

**Figure 1:**
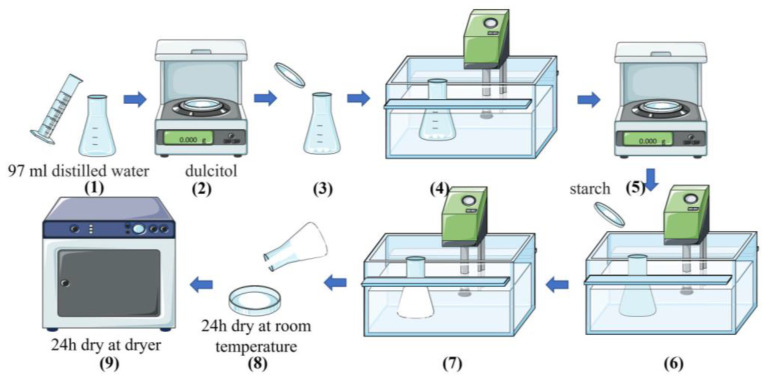
Scheme of preparation of the samples.

To obtain phase change materials, dulcitol from Sigma (Schnelldorf, Germany) (CAS: 608-66-2) with purity >99% and natural potato starch (POCh) (Gliwice, Poland) were used. All substrates were used as received. The sample preparation scheme is shown in Figure 1 and is described below.

To obtain shape-stabilized PCMs, 97 mL of distilled water was measured, and then dulcitol was weighed and added to the water. The water/dulcitol suspension was heated in a water bath (70 °C) until the sugar dissolves. Next, up to 3 g of solid substance (starch + sugar) was added to the water/dulcitol solution, and the mixture was heated until the gel formed. After that, the gel formed was poured onto Perti dishes and dried at room temperature for 24 h and at 70 °C for the next 24 h in the dryer. The composition of all the samples obtained is presented in Table 1.

Differential scanning calorimetry (DSC) measurements were performed using DSC 1 from Mettler Toledo (Greifensee, Switzerland) equipped with an intracooler. The measurement conditions were heating⟶cooling⟶heating cycle in the 25–240 °C temperature range; the heating/cooling rate was 10 °C/min under a nitrogen atmosphere (30 mL/min). After basic testing, the samples were subjected to multiple tests at different heating and cooling rates in order to find the system with the best ratio of heat stored during melting to heat released during the cold crystallization process. A step mode DSC program was applied to determine the heat stored during phase transitions [35]. Thermogravimetric measurements were made using a TGA 550 Discovery analyzer from TA Instruments (New Castle, DE, USA). Samples were measured in the temperature range of 30 to 600 °C. The heating rate was 10 K/min in a nitrogen atmosphere.

For shape-stabilization tests, the samples were heated in the dryer to 210 °C, and after 15 min, the shape and the state of the sample (solid or liquid) were assessed.

Fourier transform infrared spectroscopy (FTIR) measurements were performed using a BRUKER spectrophotometer (Bruker, Billerica, MA, USA) with a transmission adapter Vertex 70 V, operating with Opus 7.2 software. Spectra were collected in transmission mode using the KBr pastille of 4000–400 cm^−1^ after 64 scans at 4 cm^−1^.

Scanning electron microscope (SEM) observations were made using NOVA NANO SEM 200 (FEI, Hillsboro, OR, USA) with EDS. The sample was coated with a carbon layer and observed with approximately 350 and 1000×.

## 3. Results and Discussion

### 3.1. Differential Scanning Calorimetry (DSC)

**Figure 2:**
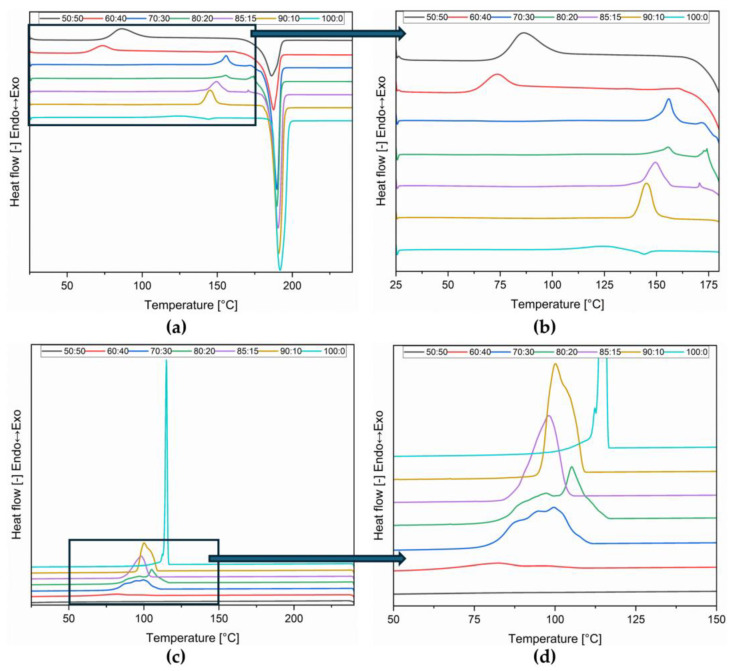
DSC curves for dulcitol/starch heating (**a**,**b**) and cooling (**c**,**d**).

To verify the material’s ability to store and release DSC heating–cooling–heating, tests were performed. The DSC curves of the second heating run were taken for analysis since the first curve is intended to remove the thermal history of the samples. The curves obtained are presented in Figure 2.

From the DSC curves, it can be seen that during heating, exothermic peaks related to the cold crystallization process appear (Figure 2a,b). Part of the material crystallizes during cooling (Figure 2c,d), and the remaining part is during the heating process. The heat of melting, crystallization, and cold crystallization are presented in Table 2.

The values achieved for a sample consisting of 100% dulcitol are similar to those achieved by other scientists under the same test conditions. Therefore, Sari et al. [36] obtained PCM based on palmitic and stearic acid esters with dulcitol. In the DSC test, the sugar alcohols melted at 187 °C, and the heat of fusion was 401.76 J/g, while upon cooling, they crystallized at 116 °C, and the heat of crystallization was 285.15 J/g. Paul et al. [19] indicated that this dulcitol is characterized by a melting point of 187 °C and a heat of phase transition of ca. 254 J/g.

Due to the largest amount of heat released during the dulcitol cold crystallization process in the dulcitol/starch 50:50 system, an attempt was made to modify the heating and cooling program for such systems to find the best conditions for cold crystallization and charging/discharging performance for long-term heat storage. Increasing the heat of cold crystallization is desired to enable the material to have as high a heat storage density. Shao et al. [37] provide the possibility of using this process for long-term thermal energy storage. The authors performed tests on mixtures of sugar alcohols, including erythritol/D-mannitol and D-dulcitol/inositol. These mixtures cannot crystallize upon cooling, and interestingly, a short exothermic peak occurred during the second heating process, which was attributed to cold crystallization. However, the area of this short exothermic peak is much smaller than that of the endothermic melting peak, suggesting that the latent heat is not fully released during cold crystallization. The fundamental mechanism of cold crystallization has not yet been elucidated [37].

**Figure 3:**
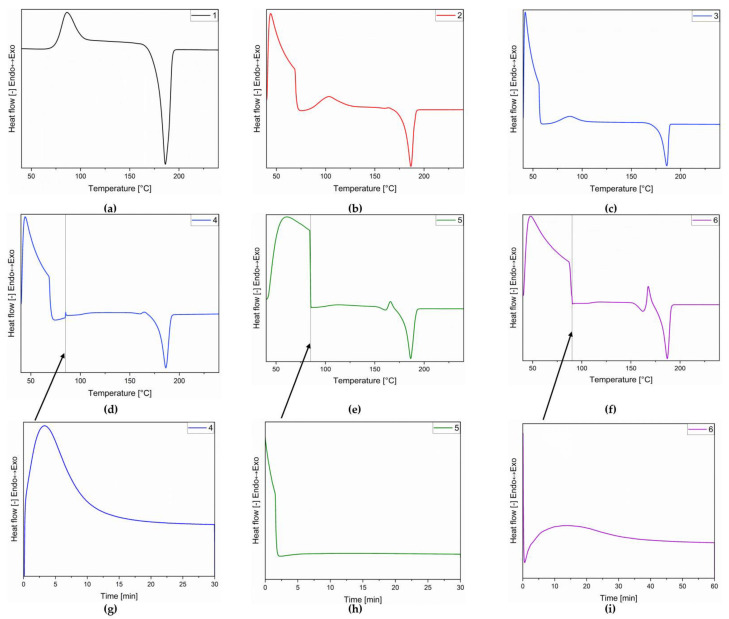
DSC curves for dulcitol/starch heating according to temperature programs 1–6, diagrams (**a**–**f**) respectively. Diagrams (**g**–**i**) are isothermal segment for program 4–6 respectively.

In order to find the most optimal charging/discharging conditions, the sample heating and cooling rates were modified during the DSC measurements. Various heating/cooling rates were tested: 2, 5, 10, 20, and 50 °C/min. Also, the colling process was with a rate of 10 °C/min or with fast cooling (ca. 80 °C). The use of rapid cooling allows sample quenching in the amorphous state and has a positive effect on the amount of heat released during the cold crystallization process; it is higher than in the case of a sample cooled at a rate of 10 °C/min. The modification in which the highest heats of melting, crystallization, and cold crystallization were achieved is shown in Figure 3 and Table 3.

The applied temperature programs are listed below:

(1) heating 10 °C/min, cooling 10 °C/min, heating 10 °C/min,

(2) heating 10 °C/min, cooling 80 °C/min, heating 10 °C/min,

(3) heating 10 °C/min, cooling 80 °C/min, heating 5 °C/min,

(4) heating 10 °C/min, cooling 80 °C/min, heating from 40 to 85 10 °C/min, isothermal segment 30 min, heating from 85 to 240 10 °C/min,

(5) heating 10 °C/min, cooling 80 °C/min, heating from 40 to 85 50 °C/min, isothermal segment 30 min, heating from 85 to 240 10 °C/min,

(6) heating 10 °C/min, cooling 80 °C/min, heating from 40 to 90 20 °C/min, isothermal segment 60 min, heating from 85 to 240 10 °C/min.

Table 3 presents the values of the individual peaks. Due to the occurrence of multiple peaks from the same type of transformation, Table 3 provides a summary of the values for each program. The charging–discharging efficiency (*η*) of the samples was counted by the following equations:

(1) $$\eta =\frac{Q_{m}}{Q_{cc}}\cdot 100$$

where *Q_m_* is the melting latent heat of the sample and *Q_cc_* is the cold crystallization latent heat of the sample [34].

Different heating rates for the material were tested, and the results showed that the lowest heating rate yielded the smallest enthalpy for cold crystallization, which explains the comparatively poor performance of the program (4). In Table 4, it can be seen that the stored and released thermal energy, revealing that, although program (6) had a slightly lower efficiency, it released the highest amount of energy. To evaluate the potential for controlled heat release, the impact of the temperature program on the enthalpy of cold crystallization was examined, and a slightly higher value was observed than in the program without this step. At present, this additional step appears to be beneficial for achieving a more controlled release of heat; however, further testing is needed to validate its effectiveness for practical applications.

The best thermal energy storage ability was achieved for the sample heated with a heating rate of 10 °C/min, then rapidly cooled to 90 °C at a rate of 80 °C/min, heated at this temperature for 60 min, and then again heated again at a rate of 10 °C/min up to 240 °C. However, the values achieved are slightly different from the results obtained for the heating program at a rate of 10 °C/min, cooled to 90 °C at a rate of 80 °C/min, and reheating at a rate of 10 °C/min. These possibilities allow you to select the appropriate program depending on the needs of the future application; a sample that had an isothermal segment during the heating stage releases heat much slower than a sample without this stage.

### 3.2. Step Mode DSC

**Figure 4:**
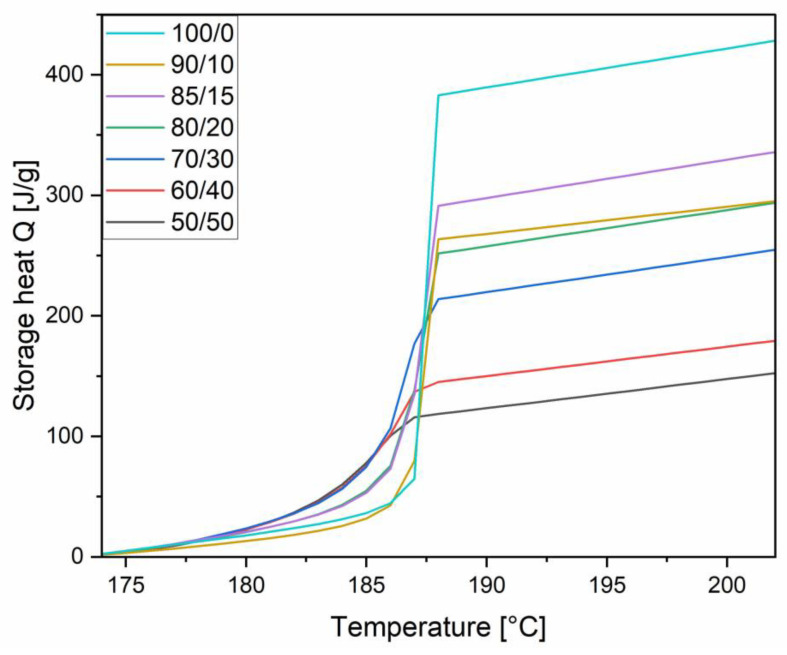
The storage capacity of dulcitol/starch PCMs.

The storage capacity of dulcitol/starch PCMs was determined using the step-mode DSC method—the obtained results have been presented in Figure 4.

A higher percentage of sugar determines a higher melting point of the material and a larger amount of stored heat. The value of stored heat for a system with 50% starch and 50% sugar alcohol content (dulcitol/starch 50/50) is three times lower than for a PCM without starch, i.e., a dulcitol without shape stabilization. The 80% dulcitol content in the shape of stabilized PCMs allowed us to achieve almost twice the value of stored energy compared to the material with 50% dulcitol. The selection of the amount of starch for the material should be determined by the most favorable ratio of energy obtained in relation to shape stabilization. At low starch contents, samples during PCM leakage tests lost their shape, and a solid–liquid transformation occurred. The aim of the current research was to obtain a material with a stable shape and a solid–solid phase transition. The best results in both shape stabilization and the stored heat amount were found for dulcitol/starch systems with mass ratios of 70:30, 80:20, and 85:15.

### 3.3. Thermogravimetric Analysis (TGA)

**Figure 5:**
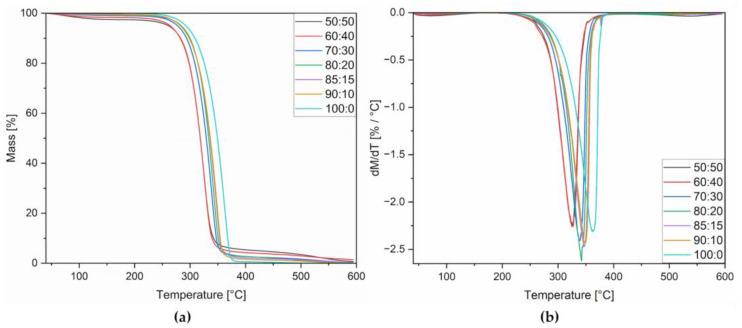
TG (**a**) and DTG (**b**) curves for dulcitol/starch systems.

The thermal stability of dulcitol/starch PCMs was investigated using the TG method—results of TG analysis are presented in Figure 5 and in Table 5.

The results obtained, as expected, indicate that starch has a negative impact on the stability of dulcitol/starch mixtures. The temperature that is important compared to thermal stability will be the melting point of the material (T_m_ = 180–190 °C). A mass loss of 1% was recorded at temperatures above 212 °C. However, the 50/50 percentage composition may turn out to be too unstable if the system in which PCM is used overheats. However, assuming no overheating of the systems, all percentage compositions are sufficiently stable. As the dulcitol content increases, the stability of the material increases. The above-mentioned shifts can be observed on the graph, where curves with higher starch content are the first to fall (the material degrades).

### 3.4. Shape Stability Test

**Figure 6:**
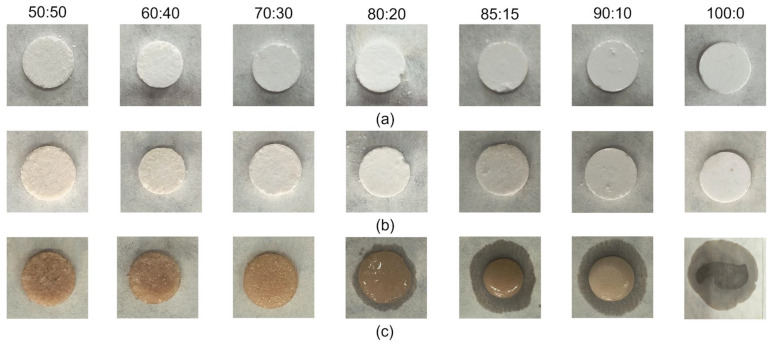
Samples subjected to shape stability analysis: (**a**) samples before analysis, (**b**) after 15 min in the dryer at 190 °C, and (**c**) after 15 min in the dryer at 210 °C.

In order to check the effect of starch on the shape stabilization of the phase change material, a shape stabilization test was performed. Samples prepared by pressing were kept in the dryer, initially for 15 min at 190 °C, then for 15 min at 210 °C (melting point of samples 187 °C—read from DSC analysis) [38,39]. The results obtained are presented as photos of the samples in Figure 6.

In Figure 6, we can see that even a small content of starch (10%) determines shape stability, but dulcitol partially melts. Starch content between 30 and 50% generates shape stability without the leakage of phase change material from the sample. Referring to the DSC results, a more favorable dulcitol/starch percentage composition is 80/20. During the test, we note the flow of material, but to a much smaller extent than in other percentage compositions with a higher sugar alcohol content.

### 3.5. Fourier Transform Infrared Spectroscopy (FTIR)

**Figure 7:**
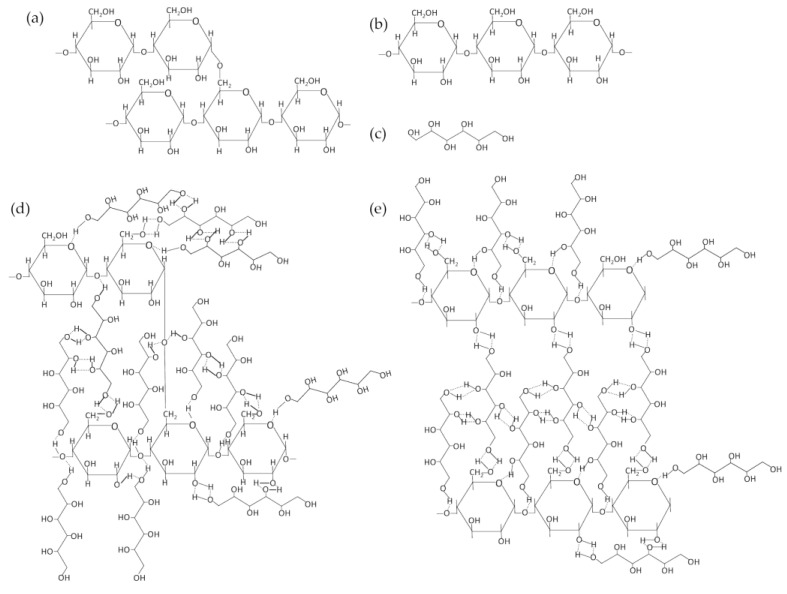
Basic chemical structural formulas of amylopectin (**a**), amylase (**b**), dulcitol (**c**) and hydrogen bond formation between amylopectin and dulcitol (**d**), amylase and dulcitol (**e**).

To analyze interactions between starch and sugar alcohols, the FTIR measurements were performed. The absorption bands presented in Table 5 are characteristic of sugar alcohols and starch. Starch consists of repeating 1,4-α-D-glucopyranosyl units and is typically a mixture of two components: linear (amylose) and branched (amylopectin). The linear component, amylose, is the minor constituent, which usually comprises 20 to 30% of starch with a molecular weight of several hundred thousand [40]. Hydrogen bonding has been theorized to play a significant role in starch gelatinization for a long time. This process involves the swelling and eventual rupture of starch granules when heated in excess water, resulting in the formation of a gel. Initial theories suggested that starch gelatinization occurred due to hydrogen bonds forming between the hydroxyl groups of starch and water molecules [41]. Sun et al. [42] discovered that amylose can form strong, elastic gels with the addition of various polyols. The possible interactions are shown in Figure 7.

The absorption bands from the new bonds do not appear in the FTIR spectra. This analysis allows us to conclude that hydrogen bonds are formed between starch and dulcitol, which in the spectrogram may be characterized by slight shifts of absorption bands involved in the formation of hydrogen bonds relative to the starting material.

**Figure 8:**
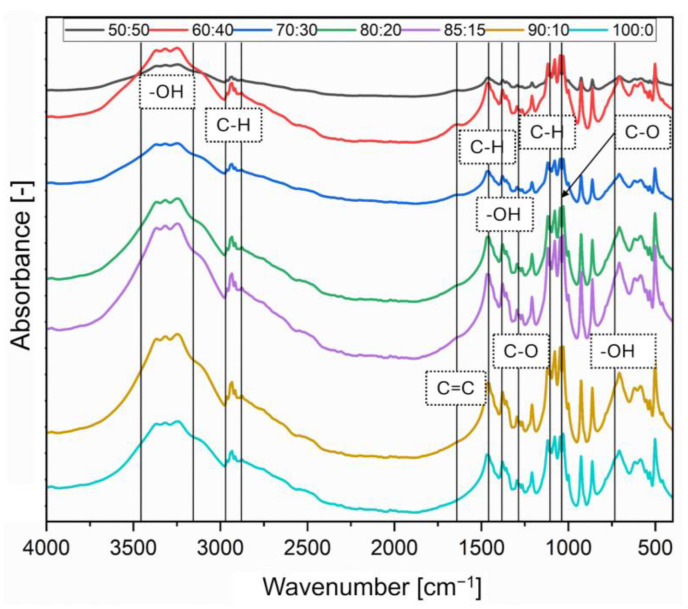
FTIR spectra of dulcitol/starch systems.

In the scheme proposed in Figure 7, the -OH groups in both compounds will behave as proton donor–proton acceptor groups. Only in the absorption bands for this bond are there minimal variations between the obtained samples. Figure 8 and Table 6 describe all marked groups characteristic of dulcitol. Table 7 shows the differences in the absorption bands of the -OH group.

The absorption bands for percentage compositions 50:50 and 60:40 are the most shifted towards the bands of the -OH group in starch (3430 cm^−1^) [41]. These slight shifts indicate that the -OH hydroxyl groups will be characterized by donor-acceptor interactions.

### 3.6. Microscopic Investigations

**Figure 9:**
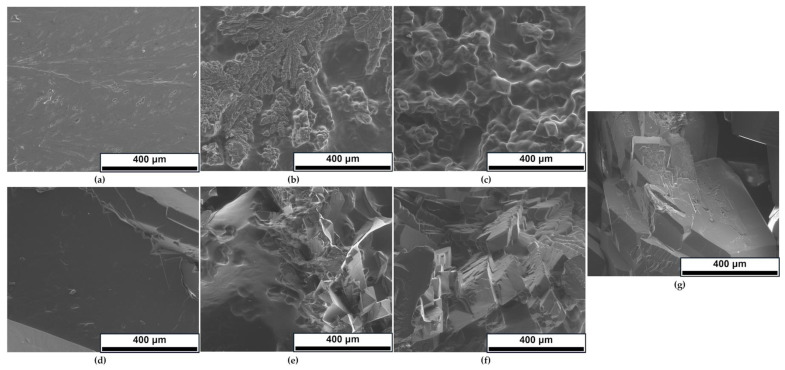
SEM microphotographs (350×) of the obtained dulcitol/starch PCMs with different mass ratios: (**a**) 50:50, (**b**) 60:40, (**c**) 70:30, (**d**) 80:20, (**e**) 85:15, (**f**) 90:10, (**g**) 100:0.

SEM microscopic observations were taken to analyze the effect of starch on the microstructure of dulcitol/starch PCMs and their crystalline structure. The obtained microphotographs are shown in Figure 9.

As the sugar alcohol content, which is the crystalline phase, increases, crystalline structures can be distinguished in the microstructure of the sample. In samples containing 50:50 starch/sugar, it is difficult to distinguish the crystalline phase. At 40:60 (b) and 30:70 (c) starch/dulcitol, the direction of crystallization of the materials becomes more apparent as the dulcitol content increases. Since dulcitol is the crystalline component, in samples with higher sugar content, characteristic crystallites are visible for the unmodified sample (g—100% dulcitol). In SEM images, starch typically appears as round, sphere-like shapes, as noted in the literature [48,49]. However, in Figure 9, these shapes are not visible, probably because all starch has undergone gelatinization. In Figure 9b,c, the rounded shapes observed in the material can be attributed to the higher starch content.

In order to complement the SEM analysis, the composition of the material purity was also tested; the results were analogous for all percentage compositions of the sample. According to EDX analysis, samples contain 71.2% and 28.8% C and O, respectively.

**Figure 10:**
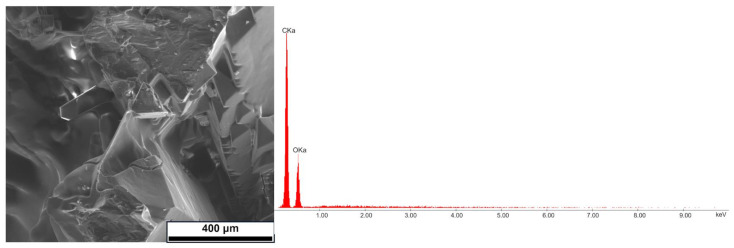
SEM (1000×) microstructural observations and EDX analysis on sample dulcitol/starch with mass ratio 85:15 *w*/*w*.

One of the results is shown in Figure 10. Carbon and oxygen were marked in the material.

**Figure 11:**
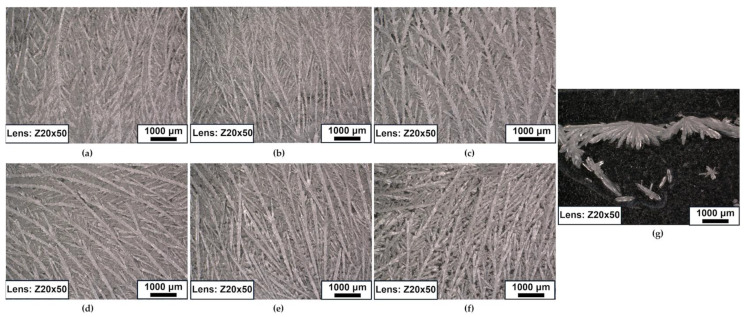
Optical microscopy (50×) microstructural observations of the obtained dulcitol/starch PCMs with different mass ratios: (**a**) 50:50, (**b**) 60:40, (**c**) 70:30, (**d**) 80:20, (**e**) 85:15, (**f**) 90:10, (**g**) 100:0.

Photos shown in Figure 11 were taken after sample preparation to check the crystallization of individual dulcitol:starch compositions. As the content of dulcitol in the composition increases, the number of crystalline structures in the material increases. Additionally, the analysis indicates that part of the material crystallized during the cooling phase, as the photo was taken after cooling rather than during reheating. Yazdani et al. [50] describe the crystallization process of similar sugar alcohol (erythritol) during cooling. In Figure 5, the sugar alcohol exhibits a visible direction of crystallization. Capturing images during the reheating process is currently not feasible; however, this could be a promising direction for future research on this material.

## 4. Conclusions

The use of starch as a shape stabilizer for organic phase change materials allows for low-cost stabilization. The applied tests demonstrate that starch does not negatively affect the thermal stability of the tested phase change material. Although the thermal stability is somewhat lower for samples with added starch, it remains higher than the material’s phase change temperature. Although some decrease in the thermal energy storage capacity was found, the application of starch as a shape stabilizer is necessary to avoid dulcitol leakage above the melting point. The amount of heat collected and released is lower for modified materials, but the benefit of shape stabilization opens the possibility of future modifications with nanoadditives. For materials with shape stabilization, the problem of sedimentation will be limited. Therefore, the material after the phase change should have the same properties as the starting material. If sedimentation occurred, the nanoparticles would modify the properties because they would not be present in the entire volume of the material but in one plane of the material. The dulcitol/starch 50:50 composition exhibits the highest efficiency in energy release during the cold crystallization process, particularly during sample reheating (releasing 52.90 J/g while storing the least amount of energy (126.16 J/g), but changing the temperature program allows an increase in the heat of cold crystallization to 127.6 J/g. This parameter is of utmost importance for long-term thermal energy storage. Energy release during reheating may facilitate longer storage durations compared to energy release during the cooling process. This resulted in the highest efficiency of the energy stored versus energy released during the cold crystallization process. The second highest value was observed in the sample with a dulcitol/starch ratio of 90:10, releasing 30.72 J/g during cold crystallization, with a significant portion of the heat (159.94 J/g) released during the crystallization process. The stored energy for this composition was 275.29 J/g. Most of the compositions tested exhibited a heat storage capacity exceeding 200 J/g. Additionally, the thermal stability for all compositions was higher than the phase change temperature, with only 1% mass loss occurring at temperatures above 200 °C, while the phase change occurred at a maximum of 190 °C. Starch additions of 20% or less resulted in minimal loss of shape stability and sugar melting, but the sample maintained its shape once it returned to room temperature.

## Figures and Tables

**Table 1:** Percentage composition of the produced samples.

Number of Sample/The Mass Ratio of:	Dulcitol	Starch
1	50	50
2	60	40
3	70	30
4	80	20
5	85	15
6	90	10
7	100	0

**Table 2:** Temperature and heat of melting, crystallization, and cold crystallization of dulcitol/starch (Q_m_—the heat of fusion, Q_c_—the heat of crystalization, Q_cc_—the heat of cold crystalization).

The Mass Ratio of Dulcitol/Starch	T_m_ [°C]	Q_m_ [J/g]	T_c_ [°C]	Q_c_ [J/g]	T_cc_ [°C]	Q_cc_ [J/g]
50:50	185	126.16	84	0.74	87	52.90
60:40	185	159.80	80, 95	27.18	70	21.40
70:30	190	229.89	90, 96, 100	125.38	155	21.19
80:20	190	234.71	97, 105	152.81	160	10.86
85:15	190	265.25	98	147.34	150	29.70
90:10	190	275.29	100, 102	159.94	145	30.72
100:0	190	341.35	112, 115	251.71	0	0.00

**Table 3:** DSC results dulcitol/starch 50:50 for different temperature measuring programs.

Temeprature Program	T_m1_ [°C]	Q_m1_ [J/g]	T_m2_ [°C]	Q_m2_ [J/g]	T_cc1onset_ [°C]	T_cc1peak_ [°C]	T_cc1endset_ [°C]	Q_cc1_ [J/g]	T_cc2onset_ [°C]	T_cc2peak_ [°C]	T_cc2endset_ [°C]	Q_cc2_ [J/g]	T_cc3onset_ [°C]	T_cc3peak_ [°C]	T_cc3endset_ [°C]	Q_cc3_ [J/g]
1	-	0.00	185	120.02	76	86	105	110.76	-	-	-	0.00	-	-	-	0.00
2	-	0.00	185	130.44	70	88	106	127.6	-	-	-	0.00	-	-	-	0.00
3	-	0.00	186	125.18	83	103	128	124.72	-	-	-	0.00	-	-	-	0.00
4	-	0.00	179	123.96	85	85	85	25.69	98	119	159	24.36	161	165	168	2.45
5	160	1.93	185	120.42	85	85	85	78.70	93	114	156	35.42	163	166	169	7.98
6	162	14.8	186	120.71	90	90	90	96.01	106	119	153	16.92	166	168	171	17.87

**Table 4:** Comparison of the heat of melting, the heat of cold crystallization, and the heat transfer efficiency of the material.

	*Q_m_* [J/g]	*Q_cc_* [J/g]	*η* [%]
1	120.02	110.76	92.28
2	130.44	127.60	97.82
3	125.18	124.72	99.63
4	123.96	52.50	42.35
5	122.35	122.10	99.80
6	135.51	130.80	96.52

**Table 5:** Thermal stability of dulcitol/starch systems.

Samples Dulcitol/Starch	T_1%_ * [°C]	T_2%_ [°C]	T_3%_ [°C]	T_5%_ [°C]	T_10%_ [°C]	T_50%_ [°C]	T_DTGmax_ [°C]	Char Residue at 600 °C [%]
50:50	212	246	257	272	290	319	326	0.644
60:40	229	249	259	270	285	319	327	1.411
70:30	243	260	269	280	295	330	338	0.229
80:20	245	266	275	286	301	334	343	0.137
85:15	249	265	274	285	300	336	347	0.247
90:10	249	264	272	284	299	337	349	0.115
100:0	258	272	281	292	309	349	362	0.057

* The values T_1%_, T_2%_, T_3%_, T_5%_, T_10%_ and T_50%_ represent the temperatures at which 1%, 2%, 3%, 5%, 10% and 50% mass loss occur, respectively.

**Table 6:** Description of characteristic bands for dulcitol/starch systems.

Wavenumber [cm^−1^]	Chemical Bond
~620	O-H_oop_ out of plane bending mode (weak and broad band) [43]
~700	CH_2_ rocking vibration [43]
862	-CH_3_ [44]
~928	O-H bending [45]
1030	C-O stretching [45]
1047	C-OH stretching vibration, C-O deformation [45]
1078	CH_3_ rocking [45]
1119	C-O (carbohydrates) [44]
1209	C-C stretching [45]
1265	C-O stretching [46]
1296	
1358	O-H bending [45]
1379	C-H bending, C-H stretching in CH3 [45]
1468	CH_2_, (bend) [45]
1639	C=C (stretch) [45]
2877	C-H, alkanes, sp^3^ [47]
2916	
2933	
3246	O-H stretching (OH groups) [45]
3317	
3367	

**Table 7:** Description of characteristic -OH bands.

Samples Dulcitol/Starch	-OH Wavenumber [cm^−1^]		
50:50	3247	3315	3380
60:40	3249	3325	3379
70:30	3248	3313	3363
80:20	3246	3323	3369
85:15	3247	3326	3360
90:10	3247	3319	3368
100:0	3246	3324	3362

## Data Availability

Data are contained within the article.
